# Revealing the differential protein profiles behind the nitrogen use efficiency in popcorn (*Zea mays* var. *everta*)

**DOI:** 10.1038/s41598-022-05545-9

**Published:** 2022-01-27

**Authors:** Shahid Khan, Vitor Batista Pinto, Antônio Teixeira do Amaral Júnior, Gabriel Moreno Bernardo Gonçalves, Caio Cézar Guedes Corrêa, Fernando Rafael Alves Ferreira, Guilherme Augusto Rodrigues de Souza, Eliemar Campostrini, Marta Simone Mendonça Freitas, Marlene Evangelista Vieira, Talles de Oliveira Santos, Valter Jário de Lima, Samuel Henrique Kamphorst, José Francisco Teixeira do Amaral, Freddy Mora-Poblete, Gonçalo Apolinário de Souza Filho, Vanildo Silveira

**Affiliations:** 1 Laboratório de Melhoramento Genético Vegetal, Centro de Ciências e Tecnologias Agropecuárias, Universidade Estadual do Norte Fluminense Darcy Ribeiro (UENF), Av. Alberto Lamego, 2000, Campos dos Goytacazes, Rio de Janeiro, 28013-602 Brazil; 2 Laboratório de Biotecnologia, Centro de Biociências e Biotecnologia, Universidade Estadual do Norte Fluminense Darcy Ribeiro (UENF), Av. Alberto Lamego, 2000, Campos dos Goytacazes, Rio de Janeiro, 28013-602 Brazil; 3 Unidade de Biologia Integrativa, Setor de Genômica e Proteômica. Centro de Biociências e Biotecnologia, Universidade Estadual do Norte Fluminense Darcy Ribeiro (UENF), Av. Alberto Lamego, 2000, Campos dos Goytacazes, Rio de Janeiro, 28013-602 Brazil; 4 Laboratório de Fitotecnia-Setor de Nutrição Mineral de Plantas, Centro de Ciências e Tecnologias Agropecuárias, Universidade Estadual do Norte Fluminense Darcy Ribeiro (UENF), Av. Alberto Lamego, 2000, Campos dos Goytacazes, Rio de Janeiro, 28013-602 Brazil; 5 Laboratório de Melhoramento Genético Vegetal-Setor de Fisiologia Vegetal, Centro de Ciências e Tecnologias Agropecuárias, Universidade Estadual do Norte Fluminense Darcy Ribeiro (UENF), Av. Alberto Lamego, 2000, Campos dos Goytacazes, Rio de Janeiro, 28013-602 Brazil; 6 Departamento de Engenharia Rural, Centro de Ciências Agrárias, Universidade Federal do Espírito Santo (UFES), Alto Universitário, s/nº, Alegre, Espírito Santo 29500-000 Brazil; 7 Instituto de Ciencias Biológicas, Universidad de Talca, 1 Poniente 1141, 3460000 Talca, Chile

**Keywords:** Abiotic, Plant breeding

## Abstract

We investigated the proteomic profiles of two popcorn inbred lines, P2 (N-efficient and N-responsive) and L80 (N-inefficient and nonresponsive to N), under low (10% of N supply) and high (100% of N supply) nitrogen environments, associated with agronomic- and physiological-related traits to NUE. The comparative proteomic analysis allowed the identification of 79 differentially accumulated proteins (DAPs) in the comparison of high/low N for P2 and 96 DAPs in the comparison of high/low N for L80. The NUE and N uptake efficiency (NUpE) presented high means in P2 in comparison to L80 at both N levels, but the NUE, NUpE, and N utilization efficiency (NUtE) rates decreased in P2 under a high N supply. DAPs involved in energy and carbohydrate metabolism suggested that N regulates enzymes of alternative pathways to adapt to energy shortages and that fructose-bisphosphate aldolase may act as one of the key primary nitrate responsive proteins in P2. Proteins related to ascorbate biosynthesis and nitrogen metabolism increased their regulation in P2, and the interaction of l-ascorbate peroxidase and Fd-NiR may play an important role in the NUE trait. Taken together, our results provide new insights into the proteomic changes taking place in contrasting inbred lines, providing useful information on the genetic improvement of NUE in popcorn.

## Introduction

Nitrogen (N) is one of the most important and required minerals for plant growth and development, directly impacting crop yields^1^. The consumption of fertilizer is constantly increasing worldwide, and the rate of N fertilizer use has increased by approximately eightfold since 1960^2^. Approximately 60% of global N fertilizer is used for the worldwide production of rice, wheat, and maize^3^.

Synthetic N fertilizer supplementation in soils has adverse effects on the environment and climate^4^. In cereal crops, such as *Triticum aestivum* L.; *Zea mays* L.; *Oryza sativa* L.; *O. glaberrima* Steud.; *Hordeum vulgare* L.; *Sorghum bicolor* (L.) Moench; *Pennisetum glaucum* (L.) R. Br.; *Avena sativa* L.; and *Secale cereale* L.), approximately 67% of N fertilizer is not absorbed and used by plants (assuming fertilizer-soil equilibrium) and lost, mostly as nitrous oxide, through gaseous plant emission, leaching, soil denitrification, surface run-off, volatilization, contributing to atmospheric greenhouse gases and environmental pollution^5^. For these reasons, the development of cultivars that maintain the same or superior crop yields, but require less N input, is necessary to supply food in a sustainable way.

Breeding for nitrogen use efficiency (NUE) is an economically and environmentally sustainable goal in the Organization of the United Nations. To increase the NUE of crops, it is essential to understand the mechanisms of N assimilation (N uptake) and remobilization (N utilization) during the plant life cycle^6^. N absorption and utilization in the plant are governed by two types of nitrogen transporter genes: (1) nitrate transporter (NRT) genes that take up N in the form of nitrate (NO_3_^−^) and (2) ammonium transporter (AMT) genes that take up N in the form of ammonium (NH_4_^+^).

NO_3_^−^ uptake generally involves two types of transport systems, one involving a high-affinity transport system (HATS) activated during low nitrate availability, and the other involving a low-affinity transport system (LATS) activated during high nitrate availability^7,8^. The *NRT1.1* gene acts as a dual affinity nitrate transporter^9^, and the LATS nitrate transporters previously described in maize roots comprise *ZmNRT1.1A, ZmNRT1.1B*, and *ZmNRT1.2*, while the HATSs are *ZmNRT2.1* and *ZmNRT2.2*^9^*.* NRT transports nitrate to the cytosol and is reduced to nitrite (NO_2_^−^) through nitrate reductase (NR) action. Then, nitrite is transferred to plastids and reduced to NH_4_^+^ via nitrite reductase (NiR). NH_4_^+^ resulting from nitrate or direct ammonium uptake through AMT is assimilated into N-containing compounds via the glutamine synthetase (GS)/glutamine-2-oxoglutarate aminotransferase (GOGAT) cycle, and then further assimilated for N metabolism through asparagine synthetase (ASN), glutamate dehydrogenase (GDH), and aspartate aminotransferase (AAT)^10–12^.

In maize, NUE is a quantitative trait that can be portioned into nitrogen recovery efficiency (NRE) and nitrogen internal efficiency (NIE)^13^. The first is related to the recovery of N from applied N fertilizer by the aerial part, and the other is related to the ability of plants to transform N absorbed into grains^14^. Integrating agronomical and physiological measurements with molecular data, provides a better understanding of the mechanisms underlying maize NUE. Proteomics contributes to the comprehension of the complex regulatory networks involved in the important phenotypic traits (i.e. nutrient perception and utilization)^15,16^.

Some earlier studies have described the accumulation of leaf protein in cereal crops under different N-treatments^17–19^, and to our knowledge, high-throughput quantitative proteomics has not yet been performed in popcorn to study the mechanisms underlying the NUE trait. The identification of these components and the understanding of their regulations, provide important tools to develop breeding strategies based on marker-assisted selection and reverse genetics approaches to explore gene function^20^. For these reasons, proteomics appears to be an important strategy to unravel the processes controlling popcorn NUE.

Here, we present a comparative proteomic analysis of two contrasting popcorn inbred lines (P2: N-efficient and N-responsive; L80: N-inefficient and nonresponsive to N), associated with agronomic and physiological measurements under different N applications. These results provide novel insights into the cellular level of popcorn NUE, which could lead the development of new breeding strategies for more efficiently using nitrogen.

## Results

**Figure 1:**
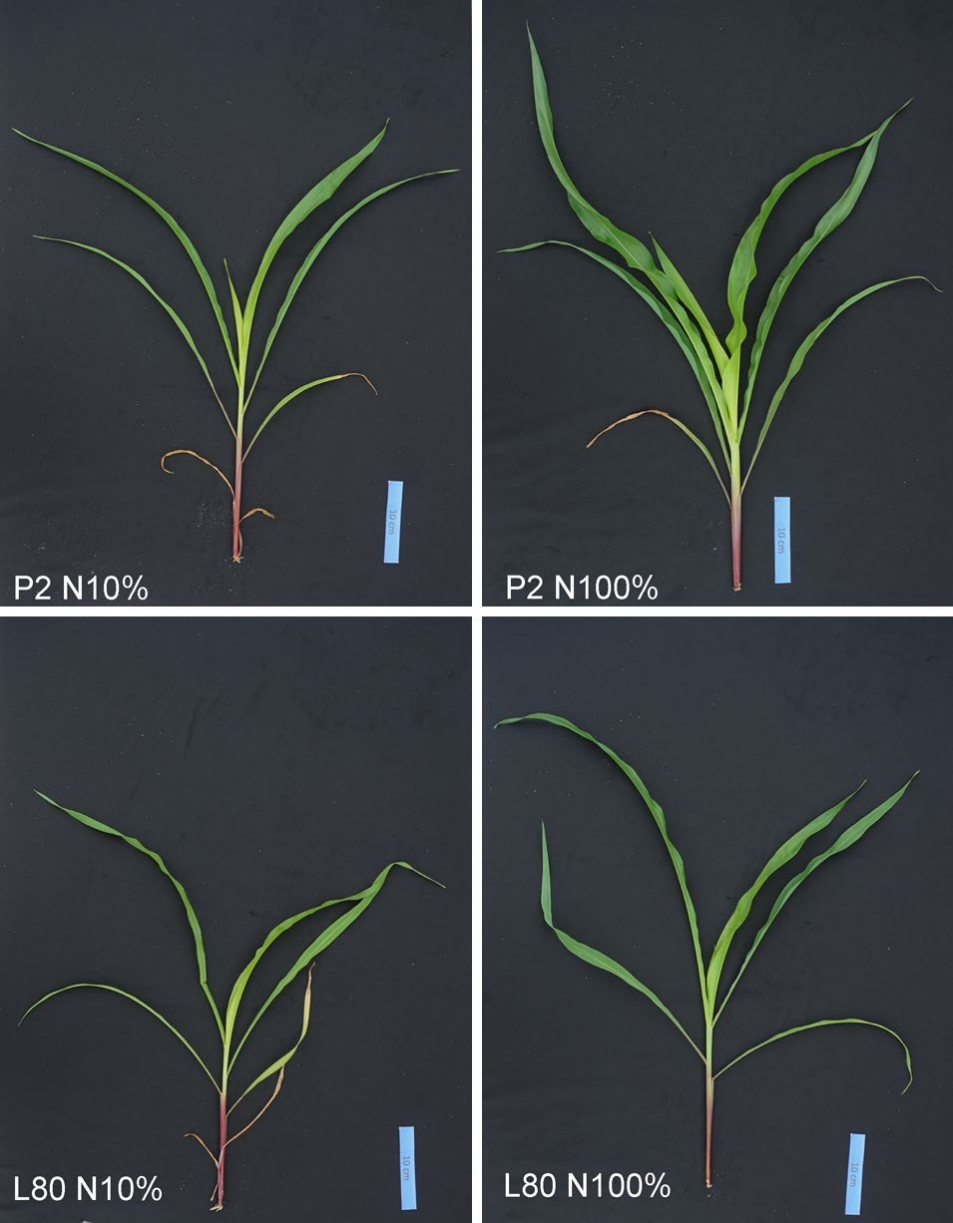
Phenotype of N-efficient (P2) and N-inefficient (L80) inbred lines at the V6 stage under low (N10%) and high (N100%) nitrogen supply.

The N supply applied in both NUE-contrasting inbred lines presented clear phenotypic differences in plant growth and leaf development (Fig. 1).

The N-efficient inbred line (P2) presented higher means for most agronomical traits and N content in comparison to the N-inefficient line (L80) at both N levels (Table 1 and Supplementary Table S1). At low N supply, P2 presented superior means for most of the traits, and the same behavior was visualized for leaf and stem dry weight and N content at high N supply (Table 1). Additionally, at low N levels, the inbred lines presented the same root dry weight and root N content (Table 1). The inbred line P2 presented a satisfactory performance independent of N dosage; however, the increment of N increased the means in L80 (Supplementary Table S1).

**Table 1:** Nitrogen content and growth-associated traits of two contrasting inbred popcorn lines under low (N10) and high (N100) nitrogen levels.

Nitrogen supply	N10		N100	
Genotype	P2	L80	P2	L80
Plant height (cm)	20.84 A	15.97 B	21.85 A	20.39 A
Leaf area (cm^2^)	43.77 A	23.41 B	59.94 A	49.26 A
Leaf dry weight (g)	1.41 A	0.6 B	2.46 A	1.4 B
Stem dry weight (g)	0.79 A	0.27 B	1.26 A	0.67 B
Root dry weight (g)	0.36 A	0.22 A	0.46 A	0.43 A
Leaf N content (mg)	31.33 A	12.01 B	71.09 A	40.8 B
Stem N content (mg)	12.9 A	4.44 B	31.34 A	19.18 B
Root N content (mg)	3.1 A	1.77 A	5.25 A	4.8 A

The average followed by the same capital letters between genotypes inside N-levels did not differ significantly by the Tukey test (*P* < 0.05, *n* = 4).

**Figure 2:**
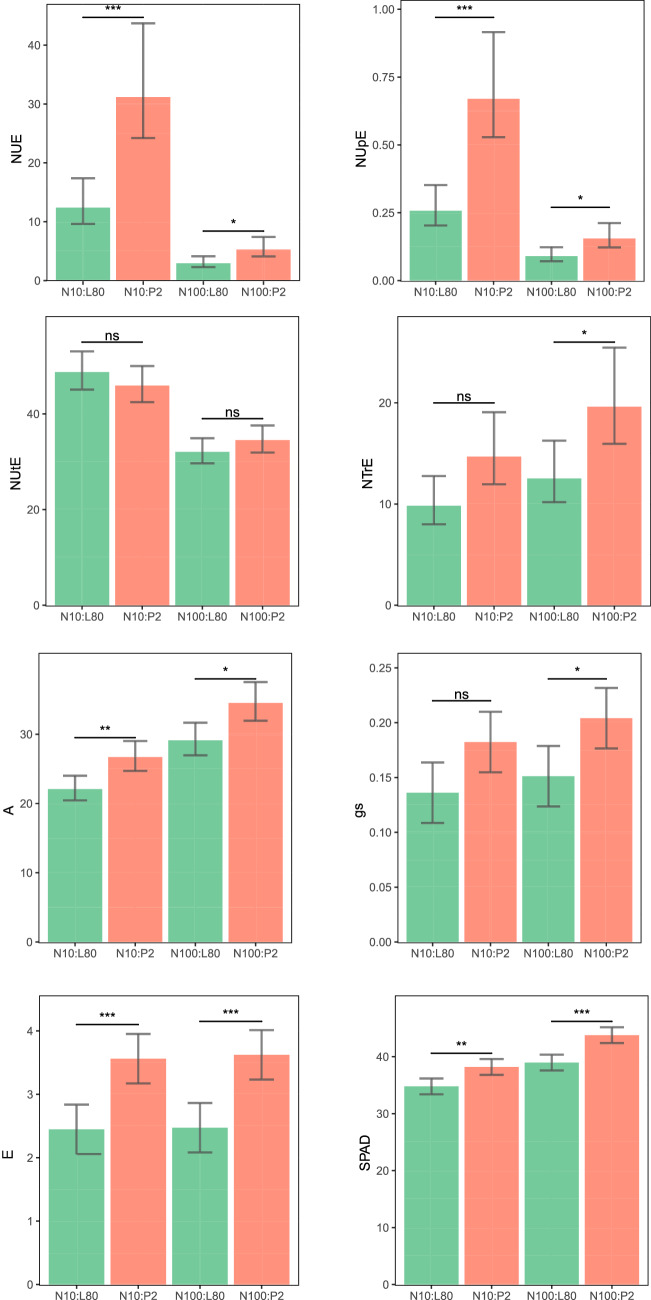
Physiological traits in popcorn inbred lines under different N supplies. *NUE* nitrogen use and efficiency (mg mg^−1^), *NUpE* nitrogen uptake efficiency (mg mg^−1^), *NUtE* nitrogen utilization efficiency (mg mg^−1^), *NTrE* nitrogen translocation efficiency (mg mg^−1^), *A* net photosynthetic rate (µmol m^−2^ s^−1^), *g*_*s*_ stomatal conductance (mmol H_2_O m^−2^ s^−1^), *E* transpiration rate (mmol m^−2^ s^−1^), *SPAD* relative chlorophyll content. Bar graphs were generated in R version 3.6.2 (https://www.r-project.org/).

The nitrogen use efficiency (NUE) and nitrogen uptake efficiency (NUpE) presented high means in P2 in comparison to L80 at both N-levels, confirming its efficiency in the absorption and use of available nitrogen (Fig. 2). The nitrogen translocation efficiency (NTrE) was significant only under high N levels, and the nitrogen utilization efficiency (NUtE) did not differ significantly between the inbred lines under either N supply (Fig. 2). At both N levels, the P2 inbred line showed superior means in all photosynthesis-related parameters, and only the stomatal conductance (g_s_) did not differ statistically between the inbred lines at the low N level (Fig. 2). Increasing the N supply, both inbred lines presented a trend to increase these photosynthesis-related traits, with the exception of transpiration rate (E), which appeared to remain unchanged (Fig. 2).

**Figure 3:**
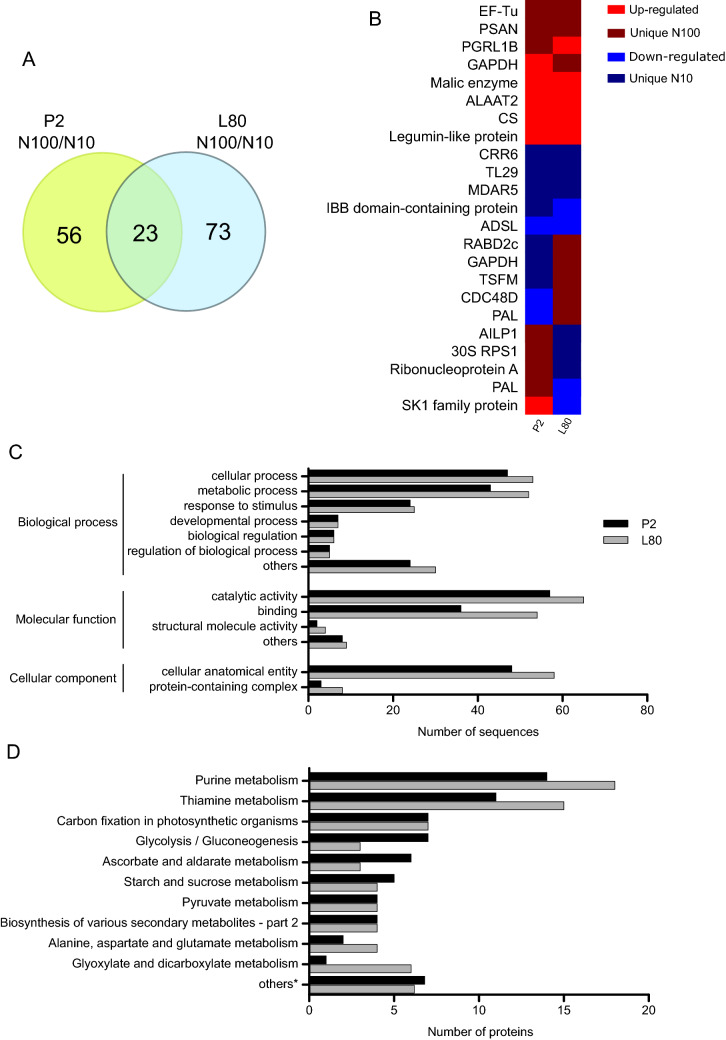
Comparative proteomic analysis of P2 and L80 under high (N100) and low (N10) N-supplies. (**A**) Venn diagram of differentially accumulated proteins (DAPs), (**B**) DAPs observed in both inbred lines, (**C**) GO enrichment analysis of GO level 2, and (**D**) KEGG enrichment analysis. *The number of DAPs were divided by ten. Heatmap was generated in R version 3.6.2 (https://www.r-project.org/).

A total of 1693 proteins were identified in maize leaves under high and low N levels (Supplementary Table S2). From this total, 79 differentially accumulated proteins (DAPs) were observed in the P2 inbred line and 96 DAPs in the L80 inbred line, both in the comparison of high N-level (N100)/low N-level (N10) (Fig. 3A; Supplementary Table S2). In P2, 23 DAPs were upregulated and 20 were downregulated, while in L80, 36 DAPs were upregulated and 26 were downregulated. We observed 22 and 19 unique proteins at low N-levels and 14 and 15 unique proteins at high N levels in the N-efficient and N-inefficient inbred lines, respectively (Supplementary Table S2). A total of 23 DAPs were observed in both comparisons (Fig. 3B).

Gene ontology (GO) analysis categorized DAPs into different groups. In the biological process category, “cellular process”, “metabolic process”, followed by “response to stimulus”, were the most representative terms in both inbred lines. The terms “catalytic activity” and “binding” in molecular function and “cellular anatomical entity” in the cellular component category were highly enriched in both inbred lines (Fig. 3C).

A KEGG pathway analysis was performed to investigate the biological function of the DAPs. The sequences from the inbred lines were mapped in several pathways. The most represented pathway was “purine metabolism”, followed by “thiamine metabolism” and “carbon fixation in photosynthetic organisms” (Fig. 3D). The pathways “glycolysis/gluconeogenesis”, “ascorbate and alderate metabolism”, and “starch and sucrose metabolism” were more representative in the N-efficient inbred line than in the N-inefficient line (Fig. 3D).

**Figure 4:**
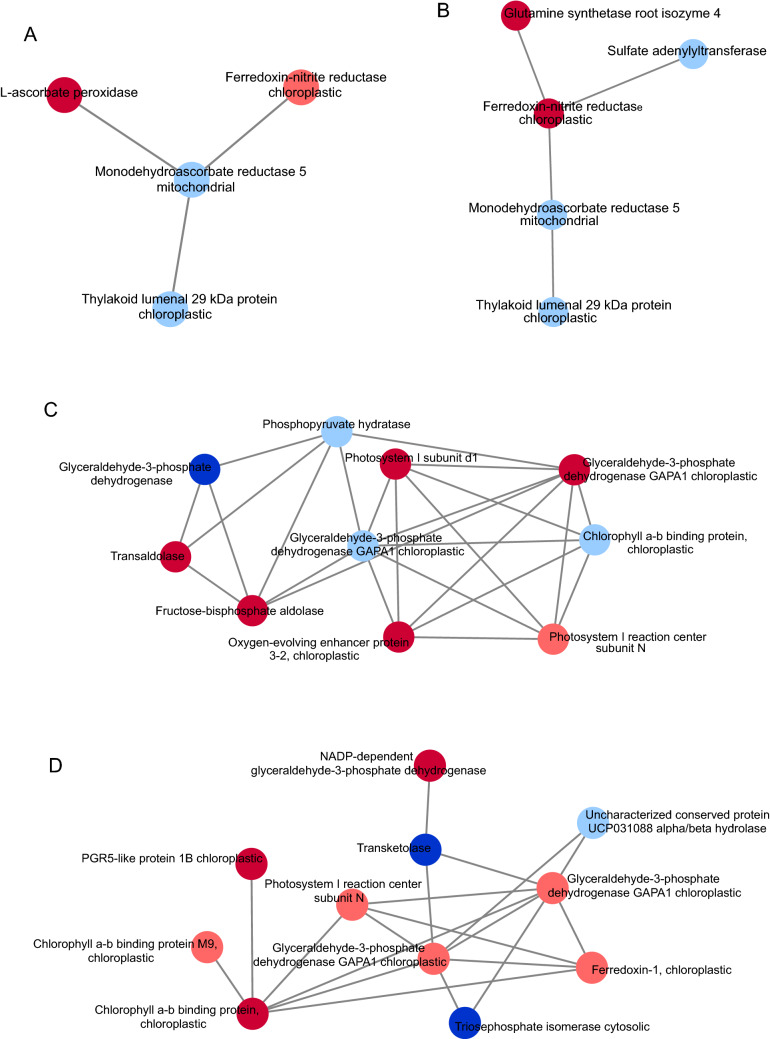
Protein–protein interaction (PPI) network between DAPs and their role in NUE. Only first neighbors are shown in the interactions. The light and dark blue ellipses represent unique proteins at low N-level and downregulated proteins, respectively. The light and dark red ellipses represent unique proteins at high N-level and upregulated proteins, respectively. (**A**, **C**) PPI network from the P2 inbred line; (**B**, **D**) PPI network from the L80 inbred line. The first degree of interaction was retrieved from STRING (version 10.5) using a minimum required interaction score of 0.7, and the network analysis performed in Cytoscape (version 3.7.1).

To visualize the interaction between the accumulated proteins, a coexpression network was constructed with potential key proteins involved in the NUE trait for both inbred lines (Fig. 4). In the P2 inbred line, ferredoxin-nitrite reductase (Fd-NiR) chloroplastic and L-ascorbate peroxidase (APX) were present at the same modulo of interaction (Fig. 4A). In the L80 inbred line, Fd-NiR interacted with glutamine synthetase (GS) root isozyme 4 (Fig. 4B). Several photosynthesis-related proteins were identified in this work (Table 2) and interacted in both inbred lines (Fig. 4C,D).

**Table 2:** Differentially accumulated proteins related to photosynthesis regulated in P2 and L80 inbred lines under high and low N supplies.

Protein ID	Description	log2 FC P2	DAP_P2	log2 FC L80	DAP_L80
K7UCS1	Photosystem I reaction center subunit N	–	Unique N100	–	Unique N100
A0A1D6GF65	Photosystem I subunit d1	0.6271	UP	–	–
P48184	Photosystem II D2 protein	1.8937	UP	–	–
A0A1D6LCA2	PGR5-like protein 1B chloroplastic	–	Unique N100	0.8158	UP
A0A1D6EY41	Chlorophyll a–b binding protein, chloroplastic	–	Unique N10	–	–
B4FTA7	PGR5-like protein 1B chloroplastic	− 0.6941	DOWN	–	–
Q41806	Oxygen-evolving enhancer protein 3–2, chloroplastic	0.6506	UP	–	–
A0A1D6IW64	Nucleoside diphosphate kinase	0.8784	UP	–	–
P27497	Chlorophyll a–b binding protein M9, chloroplastic	–	–	–	Unique N100
B4FJG1	Chlorophyll a–b binding protein, chloroplastic	–	–	0.6712	UP
A0A1D6FKP2	Protease Do-like 1 chloroplastic	–	–	1.6023	UP
A0A1D6MD76	Protease Do-like 1 chloroplastic	–	–	–	Unique N10
A0A1D6DYU7	(S)-2-hydroxy-acid oxidase	–	–	− 0.8279	DOWN
P27787	Ferredoxin-1, chloroplastic	–	–	–	Unique N100

## Discussion

Identifying efficient genotypes in N use, absorption, and translocation can optimize the cost–benefit ratio in the application of N in ecological and economic terms, avoiding the excessive use of nonrenewable resources and higher N_2_O emissions^21–23^.

The N dosage did not affect most of the growth parameters in the N-efficient inbred line (P2), and even at a high N level, the N-efficient inbred line (L80) showed inferior behavior and responsiveness, as observed previously^24,25^. Increasing the N supply from 10 to 100%, the P2 inbred line enhanced its biomass in contrast to L80^24^. Nitrogen deficiency causes decreased maize plant height^26^ and affects biomass production in rice^27^. Moreover, in a previous screening study to select popcorn inbred lines responsive to N use, P2 exhibited high expansion volume values compared to L80^28^.

Several studies have reported a decrease in the NUE and NUpE of genotypes at high doses of N^29–31^. In our data, these traits presented the same pattern when both inbred lines were grown at a high N level. Both NUE and NUpE decreased almost four times compared to the inbred lines under low N-levels. The NUtE and NTrE indexes show that although the inbred lines showed an adequate translocation capacity of N to the aerial part at high N levels, the conversion into biomass was negatively correlated with NUtE and NTrE. The content of N in all parts of the plant was much higher under high N supply than under low N supply. Experimental conduction in pots with sand may be a key factor due to the low content of available organic matter and the limitation of the association between microorganisms and roots^32,33^.

The relative chlorophyll content (SPAD) and the net photosynthetic rate are directly correlated with the efficiency of nitrogen translocation observed between the genotypes and with the increase in the leaf area and the accumulation of dry weight, given the greater carbon fixation per unit leaf area and translocation of photoassimilates to stems and roots^34–36^. Accordingly, we observed that a high N supply promoted superior values of leaf area, plant height and leaf dry weight traits in both inbred lines. In addition, the significant increase in the means of SPAD when enhancing the nitrogen doses allows the gain in the net photosynthetic rate due to the significant increases in the complex light collectors (LHCII and LHCI), responsible for allocating more energy to the formation of ATP and NADPH in the photochemical step used in the Calvin-Benson cycle for CO_2_ assimilation^37,38^.

Based on the results of the NUE, NUpE, and NUtE indexes, the inbred lines tend to be less efficient at higher N-levels. In addition, at low N levels, P2 was able to uptake and assimilate the available N. Photosynthesis-related measurements increased with increasing N supply in both inbred lines, and P2 maintained superior values. These results confirm the role of the P2 inbred line as a potential donor to NUE breeding programs.

To track the proteomic changes in popcorn leaves under different doses of N, robust shotgun label-free proteomic analysis was performed. The DAPs shared in both inbred lines (Fig. 3B) may be useful as potential targets for functional studies to understand the regulation of the popcorn response in NUE.

Shikimate kinase (SK) (A0A1D6KDZ4) was up-accumulated in P2, and at the transcriptional level, the gene SK1 was activated in response to nitrogen depletion in *Arabidopsis*^39^. Two phenylalanine ammonia lyases (PALs) (C0PL14 and A0A1D6HDL9) showed opposite regulation in both inbred lines. PAL catalyzes the conversion of phenylalanine to trans-cinnamic acid and ammonium^40^, which may serve as an N source in *Populus* × *canescens*, inducing root growth and nitrogen-use efficiency^41^. Together with other players, these proteins may contribute to the effective response in nitrogen use and efficiency in popcorn. Furthermore, differential proteins in metabolic pathways related to NUE are discussed in the following subtitle.

Plants can carry out self-regulation in response to external nutrient availability to adapt to environmental changes^18^. A primary symptom of plants under stress is energy deficit^42^, which leads to an increase in several pathways of carbohydrate metabolism and the activation of alternative pathways of glycolysis^43^. In the leaf proteome, we identified proteins responsive to the generation of energy and involved in carbon fixation in both inbred lines. In addition, proteins involved in carbohydrate metabolism were also detected.

Two glyceraldehyde-3-phosphate dehydrogenases GAPA1 chloroplastic (GAPDHs) (A0A1D6J820 and A0A1D6J815), were identified in both inbred lines (Fig. 3B, Suppl. Table S1). GAPDH is a central enzyme in glycolysis, and its overproduction slightly enhances ribulose 1,5-bisphosphate (RuBP) regeneration capacity, improving photosynthesis in rice^44^. In wheat, this enzyme was upregulated under high ammonium nitrate^45^ and N fertilizer^18^ supplies. Although it plays an oxidative signaling role, GAPDH can increase energy production by the glycolytic pathway even at low N-levels in P2.

Fructose-bisphosphate aldolase (FBA) (B4FWP0) was up-accumulated only in P2 and is involved in carbon fixation, methane metabolism and glycolysis/gluconeogenesis (Fig. 3D, Supplementary Table S2). This enzyme catalyzes fructose-1,6-biphosphate to dihydroxyacetone phosphate and glyceraldehyde 3-phosphate. At low levels of N, the expression of FBA and other enzymes involved in the Calvin cycle were repressed in *Panax notoginseng*^46^. In a high-NUE cultivar of *Brassica juncea* L., FBA was rapidly upregulated under low nitrate treatment^47^. In wheat leaves, high N levels increased the expression of FBA^18,48,49^. Our results suggest that N regulates enzymes in the glycolysis pathway to adapt to the energy shortage, promoting energy metabolism for plant development and FBA acts as one of the key primary nitrate responsive proteins in the P2 inbred line.

Alanine aminotransferase (AlaAT) promotes nitrogen uptake by the catalysis of alanine to pyruvate, allowing an increase in the reaction rates of glutamine synthetase (GS) and 2-oxoglutarate aminotransferase (GOGAT)^50^. Transgenic plants of *B. napus*, rice and barley overexpressing AlaAT improved NUE^51–53^. In *B. napus*, increased biomass and seed yield was observed when transgenic plants were grown under low N treatments, probably attributed to enhanced alanine accumulation and mobilization^51^. Based on our results, the up-accumulation of AlaAT2 (A0A1D6KCZ2) in both inbred lines may play a role in N transport, uptake and storage, and together with other regulated components, may increase the NUpE and biomass in the P2 inbred line.

Proteins involved in starch and sucrose metabolism were regulated under high N levels (Fig. 3D). These proteins may be a part of the saccharide products of carbon fixation contributing to the enhancement of the photosynthesis rate and NUtE in both inbred lines. Genes related to starch and sucrose metabolism were upregulated in the shoots of an N-efficient cotton cultivar, increasing carbon metabolism^54^. Two granule-bound starch synthase 1 proteins (GBSS 1) (A0A1D6L1M5 and A0A1D6K4M5) were detected only under low N supply in P2. According to our results, the expression of OsGBSSII was induced under N starvation, and this gene could be repressed by supplying N sources^55^.

The N supply affected the regulation of 14 proteins involved in photosynthesis (Table 2). The chlorophyll a–b binding proteins chloroplastics (CABs) belong to the light-harvesting complex (LHC) working as a light receptor, transferring excitation energy to photosystem I (PS-I) and photosystem II (PS-II)^56,57^. In maize, proteins that form the core of the photosystem I complex (PSI) increased in N treatments, suggesting the functional role of PSI in sustaining N assimilation^17^, and under low N supply, genes involved in PSI and photosystem II (PSII) were downregulated^58^. Medium- and low-NUE wheat cultivars showed strong downregulation of a photosystem II 10 kDa polypeptide family protein using transcriptomic tools^59^.

In the protein–protein interaction (PPI) network, these proteins were linked to fructose-bisphosphate aldolase and GAPDHs, supporting the hypothesis of the maintenance of proper energy balance during nitrogen supply in both inbred lines (Fig. 4C). In addition, these proteins may be related to the superior net photosynthetic rate, stomatal conductance, and relative chlorophyll content in the N-efficient inbred line. Otherwise, the PPI networks of L80 (Fig. 4D) showed several up-accumulated proteins interacting with each other, which may correspond with the lack of a decrease in photosynthesis-related measurements in L80 in comparison with P2.

Nitrogen deficiency limits the photosynthesis rate, and an enhancement of excitation energy is necessary. Ascorbate peroxidase (APX) can be induced under many biotic and abiotic stresses to protect photosynthesis^60^, playing a role by modulating reactive oxygen species levels in guard cells^61^, and ascorbate accumulation has been reported to be induced under nitrogen deficiency^62^. In P2, several proteins were up-accumulated or unique under low N supply in the ascorbate metabolism pathway, and l-ascorbate peroxidase (A0A1D6JYW6) increased its accumulation when the plants were subjected to high N levels. When the cytosolic APX1 was overexpressed in *Arabidopsis*, the dry weights of roots and shoots were higher than those of the WT under N deficiency stress^63^. The APX content decreased in barley shoots under long-term N deficiency^64^, and ascorbate metabolism was one of the main pathways associated with N stress in cucumber fruits^65^. The presence of proteins unique at low N-levels in P2 may be associated with the ascorbate synthesis promoted by nitrogen deficiency.

In the PPI network, APX was coexpressed in the same network of Fd-NiR chloroplastic (A0A1D6HL76), an important player in the NUE trait (Fig. 4A). Nitrate is absorbed from soil and reduced to nitrite by cytosolic nitrite reductase. Then, it diffuses into chloroplasts^66^ and is reduced to ammonia by Fd-Nir^67^. Ammonia is assimilated into N-containing compounds via the GS-GOGAT pathway^11^. According to our results, in proteomic analysis of maize leaves and roots, N treatments induced changes in the levels of Fd-Nir^17^. This is directly associated with the superior levels of N content in the stems of P2 plants. In L80, the interaction of Fd-NiR (K7U9U9) with a glutamate synthetase (GS) root isozyme (P38562) (Fig. 4B) suggests that under high N levels, L80 presents an apparatus able to assimilate ammonium by GS, but it is not sufficient to increase plant development under low N levels.

Finally, the identification of DAPs across popcorn inbred lines contrasting to NUE facilitates a better understanding of the genetic bases of N stress tolerance. The comparative proteomics associated with agronomical and physiological traits allowed us to identify targets involved in nitrogen transport, energy metabolism, nitrogen metabolism, and ascorbate biosynthesis. Proteins involved in carbohydrate metabolism increased the regulation at high N levels, elevating energy production in an alternative way to cope with the nutritional stress environment. The abundance of N metabolism-related genes in the N-inefficient inbred line also contributes to N stress adaptation. The DAPs coexpressed in both inbred lines can also be involved in the response to N supply acting with proteins already described for the NUE trait. In addition to understanding the dynamics of plant N efficiency and responsiveness, key proteins such as Fd-NiR, APX, GBSS 1, SK, FBA, and AlaAT may be good candidates for NUE to be explored in popcorn breeding programs.

## Methods

### Plant material and growth condition

Two popcorn (*Zea mays* var. *everta*) inbred lines—P2 (N-efficient and N-responsive) and L80 (N-inefficient and nonresponsive to N) were selected in previous experiments under high and low availability of N^24,28^. The inbred line P2 has high grain yield under low N availability and responds positively to N supply, and the inbred line L80 has reduced production under low N availability and does not respond to N supply^24,28^. These inbred lines were developed after seven cycles of self-pollination and belong to the Germplasm Bank of UENF. P2 is classified as early, temperate/tropical and was derived from Composto CMS-42 (open pollinated variety, OPV), while L80 is late, temperate/tropical and derived from the OPV Viçosa: UFV^25^, and both respond similarly in the greenhouse^24^ and in the field^25^ conditions.

The present experiment was performed in the greenhouse of Darcy Ribeiro North Fluminense State University (UENF) in January 2020 (21° 9′ 23″ S; 41° 10′ 40″ W; altitude: 14 m; temperature: 25–38 °C; relative air humidity: 70–76%). The solution for the N source was prepared according to Hoagland and Arnon^68^, with modifications. Two contrasting N doses were used: N100% (224.09 mg L^−1^ NO_3_^−^) and N10% (22.41 mg L^−1^ NO_3_^−^)^24^.

Seeds were grown in plastic pots (35 L) containing sand washed with deionized water. The plants were irrigated daily with deionized water, and nutrients were provided at the V2 stage every two days^24,69,70^. A randomized complete block design was used with two factorial treatment arrangements (2 genotypes × 2 nitrogen levels) with seven blocks, three pots per plot and one plant per pot. Three of them were considered a pool of leaves to represent biological replicates for protein sampling. The remaining blocks were used for morphological, agronomic, and physiological experiments.

### Growth measurements and N content

At the V6 stage (six fully expanded leaves), plant height (PH, cm) was measured from the sand surface to the collar of the sixth leaf. After harvesting the plant, the total plant leaf area (LA, cm^2^) was measured using a leaf area meter (Li-3100, Li-Cor).

The leaves, stems, and roots were separately wrapped in paper bags and dried in a forced air circulation oven at 72 °C for 72 h. Then, the stem dry weight (SDW, g), leaf dry weight (LDW, g), and root dry weight (RDW, g) were measured using a high-precision digital balance. N accumulation was determined as the total ammonium (NH_4_^+^) in stem-, leaf- and root-dried tissues by the method of Nessler^71^.

### Nitrogen use efficiency measurements

With the information of N content and dry weight, we calculated the N use efficiency (NUE: shoot dry weight (sum of SDW and LDW)/total N applied), N uptake efficiency (NUpE: N content in the plant (sum of SNC, LNC, and RNC)/total N applied), N utilization efficiency (NUtE-wR: shoot dry weight/N content in the plant), and N translocation efficiency (NTrE: N content in the shoot/N content in the plant)^24,72–75^.

### Physiological measurements

The net photosynthetic rate (A, μmol m^−2^ s^−1^), stomatal conductance (g_s_, mmol H_2_O m^−2^ s^−1^), and transpiration rate (E, mmol m^−2^ s^−1^) were calculated using a moveable open-system infrared gas analyzer (LI-6400, Li-COR, Lincoln, Nebraska, USA). The system incorporating a CO_2_ controller was used to set the CO_2_ concentration inside the leaf chamber to 400 μmol CO_2_ (mol[air]^−1^). The 6 cm^2^ chamber was fitted with a red–blue light source (6400-02 B), and the photosynthetically active radiation (PAR) inside the chamber was fixed at 1500 μmol m^−2^ s^−1^. The relative chlorophyll content was measured using MultispeQ V 1.0 (PHOTOSYNQ INC, East Lansing, Michigan, USA) reading SPAD-650. The data were recorded in the morning between 08:00 and 10:00 a.m. on full sunny days on the fully expanded nondetached 6th leaf at the V6 stage^76,77^.

### Protein extraction

Three biological replicates (300 mg fresh matter—FM each) consisting of the sixth fully expanded leaf at the V6 stage were collected. Each biological replicate represented a pool of leaves from each block per treatment. These leaves were macerated under liquid nitrogen, and the resulting powder was placed in 1.5 mL microtubes.

Proteins were extracted using the trichloroacetic acid (TCA)/acetone method^78^, with modifications. The samples were resuspended in 1 mL of cold extraction buffer containing 10% (w/v) TCA (Sigma Chemical Co., St. Louis, MO) in acetone with 20 mM dithiothreitol (DTT) (GE Healthcare) and vortexed for 5 min at 8 °C. The mixture was maintained at − 20 °C for 1 h and then centrifuged at 16,000*g* for 30 min at 4 °C. The resulting pellets were washed three times with cold acetone plus 20 mM DTT and centrifuged for 5 min/wash. The pellets were air-dried and resuspended in buffer containing 7 M urea, 2 M thiourea, 2% Triton X-100, 1% DTT, and 1 mM phenylmethylsulfonyl fluoride (PMSF) (Sigma-Aldrich), vortexed for 30 min at 8 °C and centrifuged for 20 min at 16,000*g*. The supernatants were collected, and the protein concentrations were determined using a 2-D Quant Kit (GE Healthcare, Piscataway, NJ, USA).

### Protein digestion

Aliquots of 100 µg of proteins/sample were used for protein digestion with trypsin. First, proteins were precipitated using a methanol/chloroform protocol to remove any detergent contaminant from samples^79^. Tryptic protein digestion (1:100 enzyme:protein, V5111, Promega, Madison, USA) was subsequently performed using the modified filter-aided sample preparation (FASP) method as described by^80^. The resulting peptides were quantified according to the A_205nm_ protein and peptide method using a NanoDrop 2000c spectrophotometer (Thermo Fisher Scientific, Waltham, USA).

### Mass spectrometry analysis

Mass spectrometry was performed using a UPLC nanoAcquity connected to a Q-TOF SYNAPT G2-Si instrument (Waters, Manchester, UK). Runs consisted of three biological replicates of 2.5 μg of peptide samples. During separation, the samples were loaded into a nanoAcquity UPLC M-Class Symmetry C18 5 μm trap column (180 μm × 20 mm) at 5 μL min^−1^ during for 3 min and then onto a nanoAcquity M-Class HSS T3 1.8 μm analytical reverse-phase column (75 μm × 150 mm) at 400 nL min^−1^, with a column temperature of 45 °C. A binary gradient for peptide elution was used with mobile phase A consisting of water (Tedia, Fairfield, Ohio, USA) and 0.1% formic acid (Sigma-Aldrich) and mobile phase B consisting of acetonitrile (Sigma-Aldrich) and 0.1% formic acid. The elution gradient started at 5% B, increasing to 43.8% up to 101.12 min, and from 43.8% B to 99% B until 105.12 min, being maintained at 99% until 109.12 min, then decreasing to 5% B up to 111.12 min and maintained at 5% B until the end of the experiment at 127.00 min. Mass spectrometry was performed in positive mode and resolution mode (mode V), with 35,000 of full width at half maximum (FWHM), and ion mobility, and in data-independent acquisition (DIA) mode. The ion mobility separation (IMS) used an IMS wave velocity of 800 m s^−1^ (HDMS^E^); the transfer collision energy increased from 19 to 55 V in high-energy mode; the cone and capillary voltages were 30 V and 3000 V, respectively; and the source of temperature was 100 °C. For time-of-flight (TOF) parameters the scan time was set to 0.5 s in continuum mode, and the mass range was 50–2000 Da. Human [Glu1] fibrinopeptide B (Sigma-Aldrich) at 100 fmol µL^−1^ was used as an external calibrant, and lock mass acquisition was performed every 30 s. Mass spectrum acquisition was performed by MassLynx software (version 4.0, Waters).

### Proteomic data analysis

Spectra processing and database search conditions were performed using ProteinLynx Global SERVER (PLGS) software (version 3.02, Waters). The HDMS^E^ analysis followed the parameters: Apex3D of 150 counts for low-energy threshold; 50 counts for elevated-energy threshold; 750 counts for intensity threshold; one missed cleavage; minimum fragment ions per peptide equal to three; minimum fragment ions per protein equal to seven; minimum peptides per protein equal to two; fixed modifications of carbamidomethyl (C) and variable modifications of oxidation (M) and phosphoryl (STY); default false discovery rate (FDR) of 1%; automatic peptide and fragment tolerance. Protein identification was performed using the *Zea mays* L. protein databank (ID: UP000007305, October 01, 2020) available on UniProtKB (www.uniprot.org). Label-free quantification analysis was performed using ISOQuant software v.1.8^81^. The parameters used were: peptide and protein FDR 1%; sequence length of at least six amino acid residues; and minimum peptide score equal to six. Samples were normalized by a multidimensional normalization process, which corrects peak intensities based on the intensity and retention time domains. The software performed the relative protein quantification based on the TOP3 method. Based on the relative abundances of uniquely assigned peptides, the abundances of shared peptides were redistributed to the respective source proteins followed by TOP3-based quantification^81^. To ensure the quality of the results after data processing, only proteins present in the three runs were accepted for differential abundance analysis. Proteins with a *p* value < 0.05 were deemed up-regulated if the log2 value of the fold change (FC) was greater than 0.60 and down-regulated if the log2 value of the FC was less than − 0.60. The functional enrichment analysis was performed using OmicsBox software 1.2.4 (https://www.biobam.com/omicsbox).

The interaction networks of DAPs used the first level of interaction retrieved by STRING version 10.5 (https://string-db.org) search. The minimum required interaction score set was 0.7 and all databases were used. The resulting protein–protein interaction network was used as an input for downstream analysis on Cytoscape version 3.7.1^82^ (https://cytoscape.org).

### Statistical analysis

A generalized linear model was performed to estimate the effect of genotypes, N levels and their interactions using the following expression:

$${Y}_{ij}={\beta }_{0}+{\upbeta }_{1}\left({N}_{i}\right)+{\upbeta }_{2}\left({G}_{j}\right)+{\upbeta }_{3}\left({N}_{i}\times {G}_{j}\right) + {\upzeta }_{ij}$$

where ${{\varvec{Y}}}_{{\varvec{i}}{\varvec{j}}}$ is the phenotype values for a given trait, considering the effects of the *i*-*th* nitrogen level and the *j*-*th* genotype; ${{\varvec{\beta}}}_{0}$ is an inherent parameter to the model (intercept model); ${{\varvec{N}}}_{i(10 and 100\% of N)}$ is the parametric vector of nitrogen fixed effects, associated with the vector ${\varvec{Y}}$ by the incidence matrix known ${{\varvec{\beta}}}_{1}$, assuming that $\mathbf{i} \sim N\left({{\varvec{\mu}}}_{{\varvec{i}}},{\mathbf{I}\otimes \sigma }_{i}^{2}\right)$, for PH, LA, LDW, SDW, RDW, LNC, SNC, RNC, gs, E, and SPAD variables, also assuming that $\mathbf{i} \sim \Gamma \left(\alpha ,\beta \right)$ for A, NTrE, NUtE, NUpE and NUE variables; ${{\varvec{G}}}_{j(L80 and P2)}$ is the parametric vector of genotype fixed effects associated with the vector ${\varvec{Y}}$ by the incidence matrix known ${{\varvec{\beta}}}_{2}$, assuming ${\varvec{j}}\boldsymbol{ }\sim \boldsymbol{ }N\left({{\varvec{\mu}}}_{{\varvec{j}}},{\mathbf{I}\otimes \sigma }_{j}^{2}\right)$ for PH, LA, LDW, SDW, RDW, LNC, SNC, RNC, gs, E, and SPAD variables also assuming that $\mathbf{j} \sim \Gamma \left(\alpha ,\beta \right)$ for A, NTrE, NUtE, NUpE and NUE variables; ${N}_{i}\times {G}_{j}$ is the parametric vector of interaction of genotype effects inside each nitrogen level, associated with the vector ${\varvec{Y}}$ by the incidence matrix known ${{\varvec{\beta}}}_{3}$, assuming that $\mathbf{i}\mathbf{j} \sim N\left({{\varvec{\mu}}}_{{\varvec{i}}{\varvec{j}}},{\mathbf{I}\otimes \sigma }_{ij}^{2}\right)$, for PH, LA, LDW, SDW, RDW, LNC, SNC, RNC, gs, E, and SPAD variables, also assuming that $\mathbf{i}\mathbf{j} \sim \Gamma \left(\alpha ,\beta \right)$ for A, NTrE, NUtE, NUpE and NUE variables; ${\upzeta }_{ij}$ is the vector of random residual effects, not captured by model effects. Means comparisons were made by adjusted Tukey’s test considering a 5% level of significance. All models were adjusted under R language.

### Consent to participate

All authors consented to participate of this research.

### Declaration of use of plant material

The popcorn seeds used in this article followed the national standards required by Ministry of Agriculture, Livestock and Supply (MAPA), agency that regulates production, processing, repackaging, storage, analysis or seed trading activities in Brazil, according to Decree Nº. 10.586, of December 18, 2020, which regulates Law Nº. 10.711, of August 5, 2003. We emphasize that none of the seeds were collected for this work, once they belong to the Germplasm Bank of UENF and come from several cycles of interpopulation recurrent selection and more recently have been evaluated for some abiotic stresses by the Laboratory of Genetics and Plant Breeding of UENF and that all works have the institution's full consent for its realization. The open pollination variety UENF-14, from which the P2 and L80 inbred lines were extracted, is registered in MAPA (registration number 29163, May 9, 2012), being the breeder Dr. Antônio Teixeira do Amaral Junior, co-author of this present work.

## Supplementary Information

Supplementary Table S1.

Supplementary Table S2.
