# Supplementary material for: Revealing the differential protein profiles behind the nitrogen use efficiency in popcorn (Zea mays var. everta)
Source: Sci Rep. 2022 Jan 27;12:1521. doi: 10.1038/s41598-022-05545-9 (PMC8795358; doi:10.1038/s41598-022-05545-9)
Supplement: Supplementary file 1 — Supplementary Table S1. [file 41598_2022_5545_MOESM1_ESM.docx]

**Supplementary Table S1**. Mean comparison of the effects of two different N levels on N content and growth in two contrasting inbred popcorn lines.

| **Genotype** | **P2** | |  | **L80** | |
| --- | --- | --- | --- | --- | --- |
| **Nitrogen supply** | **N10** | **N100** |  | **N10** | **N100** |
| Plant height (cm) | 20.84 a | 21.85 a |  | 15.97 b | 20.39 a |
| Leaf area (cm²) | 43.77 a | 59.94 a |  | 23.41 b | 49.26 a |
| Leaf dry weight (g) | 1.41 b | 2.46 a |  | 0.6 b | 1.4 a |
| Stem dry weight (g) | 0.79 a | 1.26 a |  | 0.27 a | 0.67 a |
| Root dry weight (g) | 0.36 a | 0.46 a |  | 0.22 b | 0.43 a |
| Leaf N content (mg) | 31.33 b | 71.09 a |  | 12.01 b | 40.8 a |
| Stem N content (mg) | 12.9 b | 31.34 a |  | 4.44 b | 19.18 a |
| Root N content (mg) | 3.1 b | 5.25 a |  | 1.77 b | 4.8 a |

Average followed by the same lower-case letters between N levels inside each genotype do not differ significantly by the Tukey’s test (*P* < 0.05, *n* = 4).
